# Nucleoporin 54 contributes to homologous recombination repair and post-replicative DNA integrity

**DOI:** 10.1093/nar/gky569

**Published:** 2018-07-09

**Authors:** Gonzalo Rodriguez-Berriguete, Giovanna Granata, Rathi Puliyadi, Gaganpreet Tiwana, Remko Prevo, Rhodri S Wilson, Sheng Yu, Francesca Buffa, Timothy C Humphrey, W Gillies McKenna, Geoff S Higgins

**Affiliations:** CRUK/MRC Oxford Institute for Radiation Oncology, University of Oxford, Old Road Campus Research Building, Roosevelt Drive, Oxford OX3 7DQ, UK

## Abstract

The nuclear pore complex (NPC) machinery is emerging as an important determinant in the maintenance of genome integrity and sensitivity to DNA double-strand break (DSB)-inducing agents, such as ionising radiation (IR). In this study, using a high-throughput siRNA screen, we identified the central channel NPC protein Nup54, and concomitantly its molecular partners Nup62 and Nup58, as novel factors implicated in radiosensitivity. Nup54 depletion caused an increase in cell death by mitotic catastrophe after IR, and specifically enhanced both the duration of the G2 arrest and the radiosensitivity of cells that contained replicated DNA at the time of IR exposure. Nup54-depleted cells also exhibited increased formation of chromosome aberrations arisen from replicated DNA. Interestingly, we found that Nup54 is epistatic with the homologous recombination (HR) factor Rad51. Moreover, using specific DNA damage repair reporters, we observed a decreased HR repair activity upon Nup54 knockdown. In agreement with a role in HR repair, we also demonstrated a decreased formation of HR-linked DNA synthesis foci and sister chromatid exchanges after IR in cells depleted of Nup54. Our study reveals a novel role for Nup54 in the response to IR and the maintenance of HR-mediated genome integrity.

## INTRODUCTION

Double-strand breaks (DSBs) are the most deleterious DNA lesions and are caused by endogenous reactive oxygen species derived from cell metabolism, as well as by exogenous agents such as ionising radiation (IR). If left unrepaired or misrepaired, DSBs can give rise to mutations and gross chromosomal rearrangements (1). In consequence, cells can undergo cell death, typically by mitotic catastrophe, or can survive and transmit the genetic alterations to their progeny, eventually leading to pathological conditions such as cancer (2).

The lethal effect that DSBs can have on cells is exploited in many cancer therapies, with radiotherapy being the most representative example. It is estimated that around 40% of all cancer patients are cured by radiotherapy alone or in combination with other therapeutic modalities, which stresses the importance of radiotherapy in the management of malignant diseases (3). It is recognized that the capability of cancer cells to repair DSBs and/or prevent mitotic catastrophe, i.e. intrinsic radiosensitivity, is a major limitation for radiotherapy (4). Therefore, understanding the mechanisms whereby cells deal with and survive DSBs is important for manipulating intrinsic radiosensitivity and improving radiotherapy.

Cells respond to DSBs with the coordinated activation of repair and cell-cycle control mechanisms that are integrated in the so-called DNA damage response (DDR) (5,6). There are two main DSB repair pathways in higher eukaryotes: the canonical non-homologous end joining (c-NHEJ) and the homologous recombination (HR) repair pathways. HR repair uses a homologous template, generally the sister chromatid, to restore both the integrity of the DNA molecule and the sequence in the proximity of the break. c-NHEJ repair restores the integrity of the DNA molecule by ligating the broken DNA ends, which in some instances requires prior processing of the ends and can occur between different chromosomes, leading to deletions, insertions and translocations. Whilst HR is mostly active in S and G2 phases, c-NHEJ is considered the main repair pathway throughout the cell cycle (6). Defects in these pathways can lead to a chromosomal instability phenotype characterized by increased levels of chromosome aberrations, in part as a consequence of the repair activity of more error-prone alternative pathways (alternative end joining (alt-EJ) and single strand annealing (SSA)) (1,6).

The nuclear pore complex (NPC) is emerging as an important regulator of the response to DSBs. Around 30 different proteins generically termed nucleoporins constitute this huge complex that is embedded in the nuclear envelope, and whose primary function is to regulate nucleocytoplasmic trafficking (7). Most of the evidence linking NPCs and DSB repair comes from genetic studies performed in yeast. Mutants of some nucleoporins of the inner ring (Nup170 and Nup188), the Nup84 sub-complex (Nup84, Nup120 and Nup133) and the nuclear basket (Mlp1 and Mlp2) display an enhanced sensitivity to several DNA-damaging agents, including IR (8–10). Mutations affecting the Nup84 sub-complex are synthetically lethal with mutations in components of the Rad52 epistasis group, which is involved in HR repair (9). Moreover, Nup84 and Mlp1/2 (along with another nuclear pore basket protein, Nup60) are required for appropriate SUMOylation of proteins which include the DNA damage repair factor Yku70 (10). The ubiquitylation-dependent binding of Nup60 to the Nup84 sub-complex has been shown to be required for an efficient DDR (11). The Nup84 sub-complex has also been involved in the anchoring of telomeres to the nuclear periphery, which allows relocation of DSBs to NPCs and efficient repair of sub-telomeric DSBs (12,13). Further studies in yeast have demonstrated that persistent DSBs, eroded telomeres and collapsed replication forks are actively recruited to NPCs to undergo repair (14). The Nup84 sub-complex has been shown to mediate the interaction of NPCs with persistent DSBs and collapsed replication forks, and the recruitment seems to be mediated via SUMOylation pathways (15–18).

In mammals, however, although NPCs have been shown to be permissive environments for both c-NHEJ and HR, DSBs display restricted mobility and do not migrate to the nuclear periphery (19,20). The nuclear basket—constituted by Nup153, Nup50 and Tpr—is the only NPC subcomplex with a defined role in the response to DSBs in mammals. Nup153 has been demonstrated to protect cells from DSB inducing agents by allowing the efficient nuclear import of the c-NHEJ factor 53BP1 via the importin-β pathway (21–23). Nup153 may also regulate the SUMOylation status of 53BP1 at the NPC by the SUMO protease SENP1 (24). A role in DSB repair has also been suggested for the other mammalian nuclear basket nucleoporins Nup50 and Tpr (24,25). Recent work has also shown that Nup153 and Nup50 promote the intranuclear targeting of 53BP1 to repair foci (25).

In the present study, we performed a high-throughput radiosensitivity screen with an siRNA library spanning about 10,000 genes and identified the NPC protein Nup54 as a novel determinant of the response to IR. We show that depletion of Nup54 and the molecular partners that constitute the central channel of the NPC, Nup62 and Nup58, increases the sensitivity to IR. Moreover, we demonstrate that Nup54 depletion increases IR-induced cell death by mitotic catastrophe, and specifically enhances both the length of the G2 arrest and the sensitivity of cells irradiated in S and G2 phases, as well as the formation of chromosome aberrations arising from post-replicative DNA DSBs. Furthermore, we show that Nup54 is required for the efficient repair of DSBs by HR.

## MATERIALS AND METHODS

### Cell culture

HeLa, T24, BT549, MCF7 and DU145 cell lines were acquired from the American Type Culture Collection (ATCC). HeLa cells stably expressing H2B-GFP were kindly provided by Prof. Ruth Muschel (Department of Oncology, University of Oxford). HEK293 cells with integrated EJ5-GFP and DR-GFP reporters (26) were kindly provided by Prof. Jeremy Stark (Beckman Research Institute of the City of Hope, CA). Cells were cultured in either DMEM (HeLa, HEK293 and DU145) or RPMI 1640 (T24, BT549 and MCF7) medium supplemented with 10% foetal bovine serum, at 37 °C and 5% CO_2_. All cell lines were routinely tested for mycoplasma using MycoAlert (Lonza). Cell lines grown beyond four months after purchase were authenticated by short tandem repeat profiling by the DNA Diagnostics Centre.

### Reagents

Rabbit polyclonal anti-Nup54 and Nup58 (NupL1) antibodies and mouse monoclonal anti-β-actin antibody were purchased from SIGMA. Rabbit polyclonal anti-Rad51 and mouse monoclonal anti-Nup62 antibodies were from Santa Cruz Biotechnology. Mouse monoclonal anti-γH2AX (Ser139) and anti-53BP1 antibodies were from Millipore. Rabbit polyclonal anti-Cyclin-B1 antibody was from Cell Signaling Technology. Goat anti-mouse and anti-rabbit Alexa Fluor antibodies used in fluorescence microscopy were from Invitrogen. DNA-PK inhibitor NU7741 was from Stratech. Unless otherwise stated, all other reagents were from SIGMA.

### Transfection

siRNA transfections were performed using either INTERFERin-HTS (Polyplus) or RNAiMax (Invitrogen) as transfection reagents, depending on the cell line, in a reverse transfection procedure following manufacturer instructions. Silencer Select siRNAs (Life Technologies) were used for all assays excepting the screen. The sense strand sequences of the siRNAs were as follows: si*NUP54* #1: CGAUUCAGGGUGAACUAAA; si*NUP54* #2: CUGCUGGUGUUGAUCCUAU; si*NUP54* #3: UUUAUGCAAUGAUUCUACC; si*NUP58* #1: CAAGACCAGAGGAUAGUAA; si*NUP58* #2: GCCUUGGUGGUAUAGAUUU; si*NUP58* #3: CUCUCAACAUUGACAAAUU; si*NUP62* #1: GGGCUUCAGCUUAAAGGCA; si*NUP62* #2: GCAAGAUCCUCAAUGCGCA; si*NUP62* #3: GGAGAGCCUGAUCAACAAAA; si*NUP153*: CGAAAAUCUCUCUACCGAU; si*RAD51*: GGUAGAAUCUAGGUAUGCA; si*PRKDC*: GCGUUGGAGUGCUACAACA; si*BRCA2*: GGAUUAUACAUAUUUCGCA. Unless indicated otherwise, the sequences indicated above as #1 were used for all experimental procedures. Silencer Select Negative Control No. 1 siRNA (Life Technologies) was used as negative control. The seeding density was from 150,000 to 200,000 cells per 6-well plate well, depending on the cell line. siRNAs were transfected at a concentration of 20 nM, except in the experiments where si*NUP58* and si*NUP62* were used, in which a concentration of 5 nM was used due to the considerable toxicity observed in colony formation assays at 20 nM (data not shown). Knockdown confirmation at the time of IR was routinely assessed by western-blotting and treatments were initiated 72 h after transfection.

### Colony formation assay

Cells were plated as single cell suspensions and left to attach for 4 h at 37°C and 5% CO_2_ prior to irradiation. Colonies were grown for 7–14 days, stained with crystal violet and automatically counted using a GelCount plate scanner (Oxford Optronix). The plating efficiency (PE = Average Colony Number/Cells plated) and the surviving fraction (SF = PE_IR Dose_/PE_0 Gy_) at a given IR dose was calculated. Survival data was fitted according to a linear quadratic equation and, for comparison between curves, the sensitization enhancement ratio at a surviving fraction of 0.10 (SER10) was calculated, as described previously (27). The significance of differences between curves was calculated by two-way ANOVA, with survival as the dependent variable and irradiation doses and treatment conditions as the two independent variables.

### High-throughput colony formation assay siRNA screen

Human ON-TARGETplus siRNA Library (Dharmacon) containing 9,962 siRNA pools (four strands per gene) targeting the ‘druggable’ genome was screened using a colony formation assay essentially as previously described (27). The top 240 hits were selected for a secondary screening using a human custom-made siRNA Library (Qiagen; single strand per gene) run in triplicate. For both the primary and secondary screen targets were ranked in ascending order based on the R-score ((Normalized SF – 1)/(mean absolute deviation of normalized non-targeting SF)). Rank product analysis was carried out between replicate runs for the secondary screen. The quality of the screening method determined by the dynamic range between the positive (*PRKDC* siRNA) and negative control siRNAs (non-targeting pool siRNA) was assessed by Z-factor calculation.

### Irradiation

For the screen, plates were irradiated using a Varian IX linear accelerator to a total dose of 7 Gy, as previously described (27). For the other experiments, plates were irradiated with IR at a dose rate of 1.938 Gy × min^−1^ using a GSR D1 caesium-137 irradiator (Gamma Service).

### Time-lapse microscopy

Phase contrast and green fluorescence images of live HeLa H2B-GFP cells, seeded on glass bottom plates (Ibidi) and incubated at 37°C and 5% CO_2_, were acquired with an Eclipse Ti-E microscope (Nikon) every 4 min for 48 h. H2B-GFP allowed for the observation of morphological changes in chromatin. Mitosis length was defined as the time from chromatin condensation initiation to complete karyokinesis and cytokinesis (see exception below), the latter observed by means of phase contrast. Each cell lineage was represented as a single bar corresponding to single cells within a lineage (*n* = 100), according to the following criteria: Cell lineages were classified as ‘surviving’ when all the cells within the lineage survived, and classified as ‘cell death’ when at least one cell within the lineage died. When the same type of event occurred in parallel branches within a lineage, the ones scored and represented were those initiated earlier. Cell death was sub-classified as either death in interphase or death during mitosis. Polyploidization events in the surviving lineages—either by daughter cell fusion or karyokinesis without cytokinesis—were also scored. In the case of karyokinesis without cytokinesis, for mitotic length representation, mitosis completion was defined just by karyokinesis.

### PI-exclusion and β-galactosidase assays

The propidium iodide (PI)-exclusion assay was used to assess the percentage of cells with impaired membrane permeability as an estimate of cell death. Floating and attached cells were collected together, resuspended in 50 μg/ml PI in phosphate-buffered saline (PBS) and immediately analysed by flow cytometry. Cells positive for PI staining were considered as dead cells. β-galactosidase activity was assessed by flow cytometry essentially as previously described (28). Briefly, subconfluent cells were incubated first with 100 nM bafilomycin A1 for 1 h for lysosomal alkalinization, and then with 33 μM of the β-gal substrate C_12_FDG (5-dodecanoylaminofluorescein di-β-D-galactopyranoside) for 1 h. Prior to data acquisition, 50 μg/ml PI was added as a vital dye for excluding dead cells from the analysis. The percentage of cells positive for β-galactosidase activity was calculated based on a hydrolysed C_12_FDG fluorescence intensity threshold set in untreated cells. All the flow cytometry data were acquired with a FACSCalibur flow cytometer (BD) and analysed using the Cyflogic software (http://www.cyflogic.com).

### Detection of mitotic cells in asynchronous cultures and following G1 synchronization

G1/S synchronization by double thymidine (DT) block was initiated 34 h after siRNA transfection. Cells were incubated with 2 mM thymidine for 14 h, then released for 10 h after a PBS wash and medium replacement, and incubated again with thymidine for 14 h. Immediately after exposure to IR, thymidine was washed off and 100 ng/ml nocodazole added for mitotic arrest. For both experiments in asynchronous and G1/S synchronized cultures, cells were either left untreated or exposed to 4 Gy IR 72 h after siRNA transfection. At different time points, floating and attached cells were collected together and fixed with ice-cold 70% ethanol. Fixed samples were incubated in 0.2% Triton X-100 in PBS for 15 min at 4°C for nuclear envelope permeabilization, then with a rabbit anti-human phospho-Histone 3 (Ser10) (p-H3) antibody (Cell Signalling) in 1% bovine serum albumin (BSA)/PBS for 2 h and, after a wash with 1% BSA/PBS, with a secondary anti-rabbit Alexa-488 antibody for 1 h in the dark. Finally, after a wash with 1% BSA/PBS, samples were incubated with 50 μg/ml PI and 200 μg/ml RNase in PBS for 20 min, and analysed by flow cytometry. Doublets were excluded from the analysis. p-H3-positive cells with 4n DNA content were considered as mitotic cells.

### BrdU label-chase experiment

Cells were incubated with 15 μM bromodeoxyuridine (BrdU) for 30 min and then left unirradiated or exposed to 4 Gy IR. Immediately afterwards, BrdU was removed and cells were returned to 37°C and, at different time points, floating and attached cells were collected together and fixed with ice-cold 70% ethanol. Samples were incubated with 0.1 mg/ml pepsin in 2 M HCl at room temperature in the dark for 20 min. Then, samples were incubated with mouse anti-BrdU (BD Bioscience) in 1% BSA/PBS for 2 h and, after a wash with 1% BSA/PBS, with a secondary anti-mouse Alexa-488 antibody for 1 h in the dark. Finally, after a wash with 1% BSA/PBS, samples were incubated with 50 μg/ml PI and 200 μg/ml RNase in PBS for 20 min, and analysed by flow cytometry. Doublets were excluded from the analysis. The fraction of cells irradiated in S and that were in late S/G2 at the time of fixation was determined by gating on BrdU-positive cells with 4n DNA content.

### Fluorescence microscopy

Cells grown on coverslips were fixed with 4% paraformaldehyde (PFA) for 10 min at room temperature. After two washes with PBS, samples were incubated with 1% goat serum (GS)/1% BSA/0.1% Triton X-100/PBS for 1 h at room temperature, and later with the corresponding primary antibody diluted in 1% BSA/1% GS/PBS overnight at 4°C. Following washes with PBS, samples were incubated with the corresponding fluorescent secondary antibodies and 0.5 μg/ml 4′,6-diamidino-2-phenylindole (DAPI) for 1 h, washed with PBS and mounted using Vectashield medium (Vector Laboratories). Images were randomly acquired with an Eclipse Ni-E fluorescence microscope (Nikon) using a 40× objective for nuclear 53BP1 levels (≥5 fields per sample), and a 60× objective for γH2AX and Rad51 foci assessment (>25 fields per sample). Nuclear 53BP1 levels and γH2AX and Rad51 foci number were determined using ImageJ software (29).

### Assessment of BrdU foci in G2 cells

Detection of BrdU foci after DNase treatment conditions was performed as described previously with slight modifications (30). Briefly, 1 μg/ml aphidicolin (Calbiochem) was added immediately before IR, cells were irradiated with 4 Gy and incubated with 10 μM BrdU for 6 h. Then, cells were fixed with methanol for 30 min at −20 °C and permeabilized with acetone for 1 min at −20 °C. After blocking, cells were incubated with anti-Cyclin-B1 overnight at 4°C, fixed with 2.5% PFA for 20 min and then incubated with 10 units/ml DNase I (Promega) and anti-BrdU antibody in 60 mM Tris/0.6 mM MgCl_2_/1 mM β-mercaptoethanol for 1 h at 37°C. Afterwards, samples were incubated with anti-BrdU antibody overnight at 4°C and processed for fluorescence microscopy analysis as described above for foci assessment. BrdU foci in G2 (pan-nuclear BrdU-negative/Cyclin-B1-positive) cells were scored using Imaris software (Bitplane).

### Determination of chromosome aberrations and sister chromatid exchanges

After incubation with 30 ng/ml KaryoMAX colcemid solution (Gibco) for 1 h at 37°C, cells were collected by trypsinization, incubated in Optimal Hypotonic solution (Genial Genetics) for 20 min at 37°C and fixed with ice-cold 3:1 methanol/glacial acetic acid. The cell preparation was dropped onto microscopy slides and left to evaporate completely on a metal tray at 50°C. Then, the preparations were stained with 0.5 μg/ml DAPI in PBS for 10 min, washed three times in PBS and mounted using Vectashield. Images of metaphase spreads were randomly acquired using a fluorescence microscope, with a 100× objective, and randomized for blind analysis using an ImageJ macro (29). The chromosome aberrations scored included breaks, gaps and exchanges, dichotomized into either chromatid-type or chromosome-type chromosome aberrations, as previously described (31,32). Data were presented as mean numbers of chromosome aberrations per metaphase. For estimation of the mean number of chromosome aberrations per 100 cells, the number of chromosome aberrations per metaphase was multiplied by the proportion of p-H3-positive (mitotic) cells. For this purpose, cells were also assessed by flow cytometry for p-H3 after KaryoMAX colcemid incubation as described above.

For the sister chromatid exchange (SCE) assay, 24 h after transfection cells were incubated with 10 μM BrdU for two rounds of replication (about 48 h). Colcemid was added from 8 to 12 h after 4 Gy irradiation and cells were processed as described above for the preparation of chromosome spreads. Slides were incubated for 25 min with 26.7 μg/ml Hoechst in PBS and subjected to UV light for 25 min in McIlvaine’s buffer pH 8.0 at 40°C. Then, slides were stained with 6% KaryoMAX Giemsa (Gibco) diluted in PBS and mounted in DPX. Images were acquired and randomized for analysis as described for chromosome aberrations. SCEs were scored as reciprocal discontinuities in the staining intensity of sister chromatids.

### I-SceI-based reporter assays

The I-SceI-based assays were performed according to the method described by Gunn and Stark (26). Briefly, HEK293 cells harbouring the DR-GFP (HR) and EJ5-GFP (NHEJ) reporters were transfected with siRNAs using Lipofectamine RNAiMAX (Invitrogene) as transfection reagent. Forty-eight hours later, cells were transfected with the I-SceI plasmid. Seventy-two hours after siRNA transfection GFP-positive cells were scored by FACS analysis.

## RESULTS

### Identification of central channel Nucleoporins Nup54, Nup62 and Nup58 as novel radioprotecting factors

**Figure 1.**
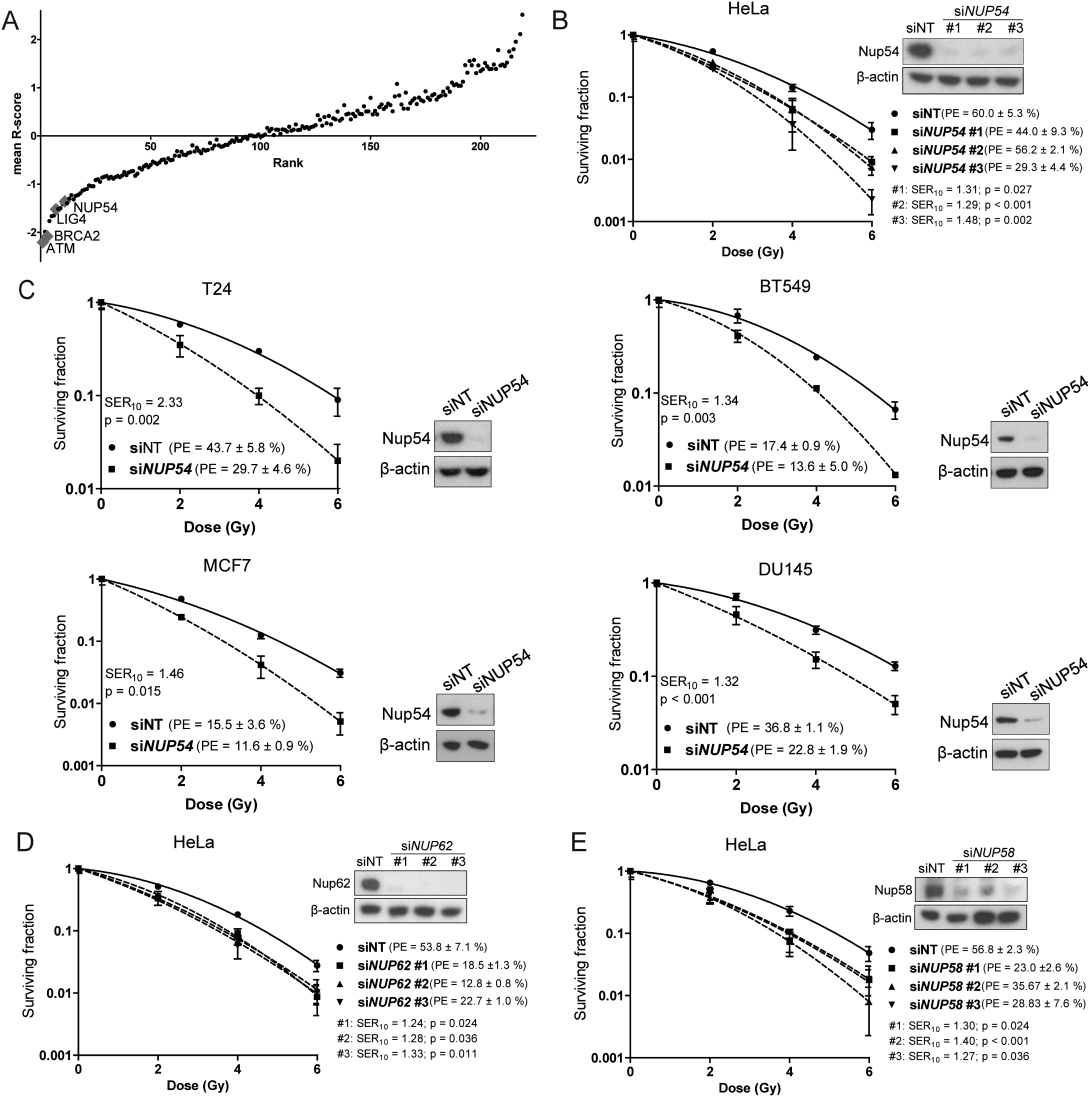
Identification of central channel Nucleoporins Nup54, Nup62 and Nup58 as novel radioprotecting factors. (**A**) Mean R-scores values for the genes in the secondary screen ordered by rank product. A negative R-score denotes radiosensitization. (**B**–**E**) CFAs performed in HeLa, T24, BT549, MCF7 and DU145 cells following siRNA transfection with the indicated siRNAs. *P*-values < 0.05 were considered significant (ANOVA; *n* = 3 technical replicates from a representative experiment repeated at least three times). Nup54 knockdown was confirmed by western blotting 72 h after transfection (β-actin level was used as a loading control).

With the aim of identifying novel determinants of radiosensitivity, we screened an siRNA library targeting 9,962 potentially druggable genes for their ability to decrease colony formation capability after IR in cervical carcinoma HeLa cells (Supplementary Excel File), following the same procedure previously implemented by our group that allowed the successful screening of the kinase subset siRNA library (27). The top 240 genes with a potential radioprotecting role were re-screened using different siRNAs, as a second confirmatory readout, and in order to increase the confidence that the results observed in the primary screen were not due to off-targeting. The identification of known radiosensitivity modulators among the top hits, including *ATM, BRCA2* and *LIG4* (Figure 1A and Supplementary Excel File), confirmed the validity of the screening method. Among the top potential radioprotecting genes, we identified the NPC protein Nucleoporin 54 (Nup54) (Figure 1A and Supplementary Excel File). Several NPC proteins have been implicated in the response to DSB, mainly in yeast (8–25,33). To the best of our knowledge, however, nothing has been described in this regard for Nup54 or any of its homologues either in yeast or in higher eukaryotes. Due to the emerging role of NPCs in the response to DNA damage and the novelty of Nup54 in this respect, we decided to further validate this finding.

In order to confirm that the effect seen in the screen was not off-target, we transfected HeLa cells with three different siRNA strands targeted against different regions of the *NUP54* transcript and assessed the colony formation capability. All three siRNAs (si*NUP54* #1, #2 and #3) caused a marked increase in the sensitivity to IR in comparison to the non-targeting siRNA (siNT) used as a control, with a concomitant strong decrease in the Nup54 protein levels, as shown by western blotting (Figure 1B). The radiosensitization induced by Nup54 knockdown (KD) was not restricted to HeLa cells. T24 (bladder), BT549 (breast), MCF7 (breast) and DU145 (prostate) cancer cells also displayed increased sensitivity to IR upon Nup54 KD (Figure 1C). Nup62 and Nup58 are the other two nucleoporins that together with Nup54 constitute the central channel of the NPC (34,35). In the screen, the library siRNAs targeting Nup62 strongly decreased colony formation in the absence of irradiation, whilst the siRNAs targeting Nup58 considerably decreased colony formation and slightly increased radiosensitivity (Supplementary Excel File and data not shown). Transfection with siRNAs from a different vendor at a lower concentration (5 nM) efficiently decreased the levels of Nup62 and Nup58 without drastically compromising viability in unirradiated cells, and caused an increase in the sensitivity to IR (Figure 1D and E). These results suggest that the central channel subcomplex of the NPC has a role in the modulation of radiosensitivity.

### Nup54 depletion increases cell death by mitotic catastrophe after IR exposure

**Figure 2.**
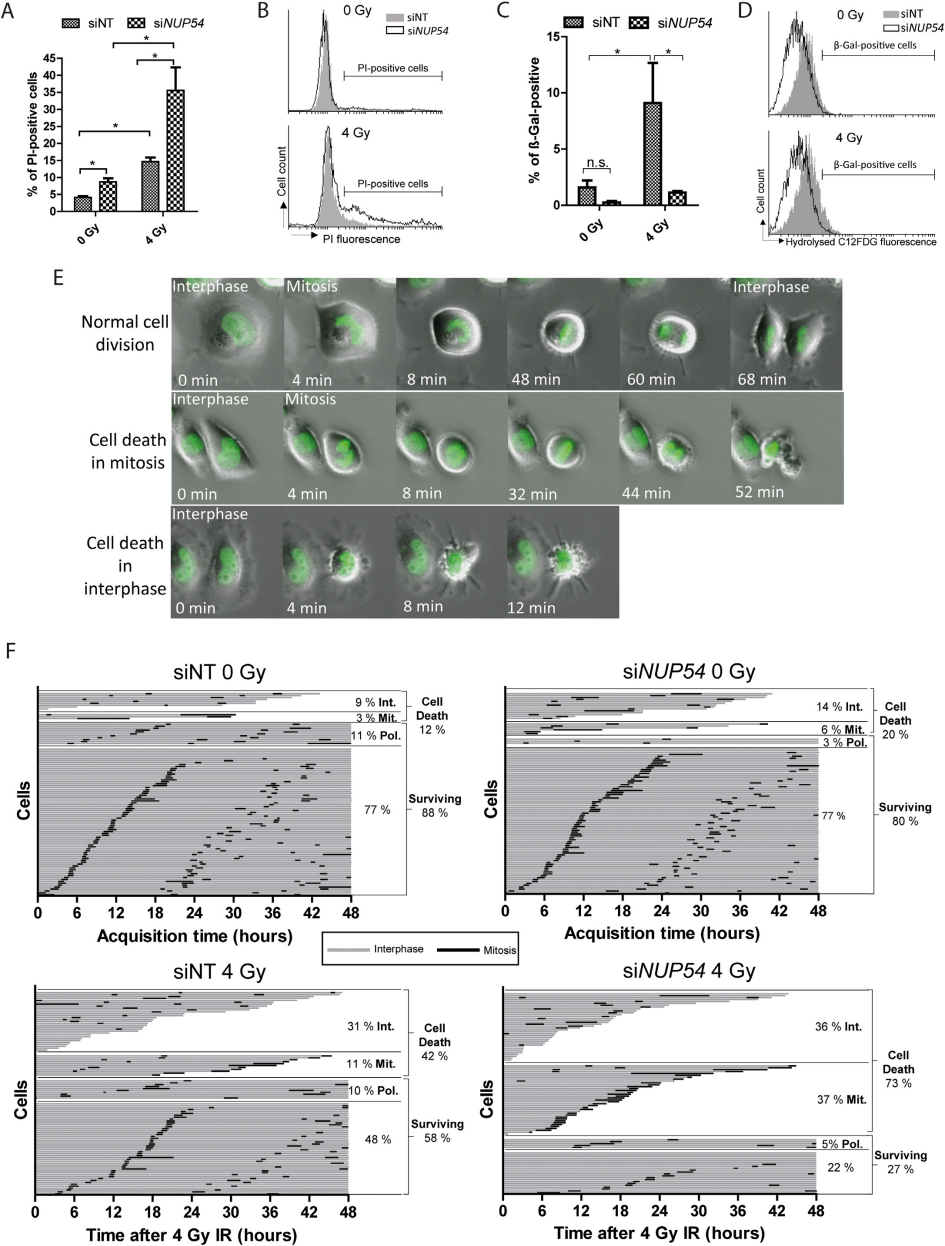
Nup54 depletion increases cell death by mitotic catastrophe after IR exposure. (**A**) Percentage of PI-positive HeLa cells measured by flow cytometry as an estimate of cell death 48 h after IR. (**B**) Histograms from a representative PI-exclusion assay. (**C**) β-gal activity assessed in HeLa cells by flow cytometry 72 h after IR. (**D**) Histograms from a representative β-gal assay. Data depicted in A and C correspond to mean ± sd of three independent experiments (*P < 0.05; two-sided *t*-test). (**E**) Time-lapse microscopy images of HeLa cells stably expressing H2B-GFP undergoing normal division, cell death in mitosis and cell death in interphase. (**F**) Graphs from a representative time-lapse microscopy experiment (*n* = 3) where each bar represents a cell lineage. The percentage of lineages surviving and undergoing cell death are shown. Cell death is sub-classified as cell death in interphase (Int.) or cell death in mitosis (Mit.). The percentage of surviving cell lineages undergoing polyploidization (Pol.) is also shown.

The loss of clonogenic potential after exposure to IR can occur via two general processes: cell death or replicative senescence (2). Impaired plasma membrane permeability is considered an unequivocal feature of any cell death modality, which can be evidenced with the use of vital dyes such as PI (36). As a first approach to determine whether the increased radiosensitivity observed upon Nup54 depletion is due to cell death, we assessed the proportion of PI-positive cells by flow cytometry 48 h after 4 Gy IR. In unirradiated cells, Nup54 KD resulted in a slight increased cytotoxicity, as evidenced by a higher proportion of PI-positive cells. After irradiation, si*NUP54*-treated cells exhibited a profoundly higher cell death induction than siNT cells (Figure 2A and B). In accordance with this, we also observed a potentiation in the induction of caspase-3/7 activity, a marker of apoptosis, in si*NUP54*-treated cells after IR (Supplementary Figure S1). Next, we explored the possible contribution of senescence to the Nup54 KD-induced radiosensitization by assessing β-galactosidase (β-gal) activity (28), 72 h after IR. Unlike siNT-treated cells, si*NUP54*-treated cells remained refractory to β-gal activity induction by IR (Figure 2C and D), suggesting that senescence does not contribute to Nup54 KD-induced radiosensitization. The increased cell death observed in Nup54-depleted cells could account for this apparent absence of β-gal activity induction.

IR-induced cell death can take place during interphase or via mitotic catastrophe (2). Mitotic catastrophe is defined as a cell death type that occurs either during or shortly after aberrant mitosis (2,36). Whilst there are no specific biochemical markers for mitotic catastrophe, this cell death modality can be assessed using live-cell imaging (2,36). In order to have a more detailed view of the cell death processes underlying Nup54 KD-induced radiosensitization, we tracked by time-lapse microscopy the fate of individual HeLa cells stably expressing GFP-tagged Histone 2B (H2B-GFP). H2B-GFP allows chromatin visualization and, therefore, the discrimination between interphase and mitosis (Figure 2E). In agreement with our flow cytometry data, unirradiated si*NUP54*-treated cells showed slightly higher cell death rates than siNT-treated cells (Figure 2F). IR induced an increase in cell death in both conditions, but to a greater extent in si*NUP54*-treated cells, with mitotic cell death accounting for most of this difference (Figure 2F). Altogether, these results suggest that Nup54 KD increases cell death after IR, mostly via mitotic catastrophe.

### Nup54 depletion causes a prolonged G2 arrest specifically in cells irradiated in S and G2 cell-cycle phases

**Figure 3.**
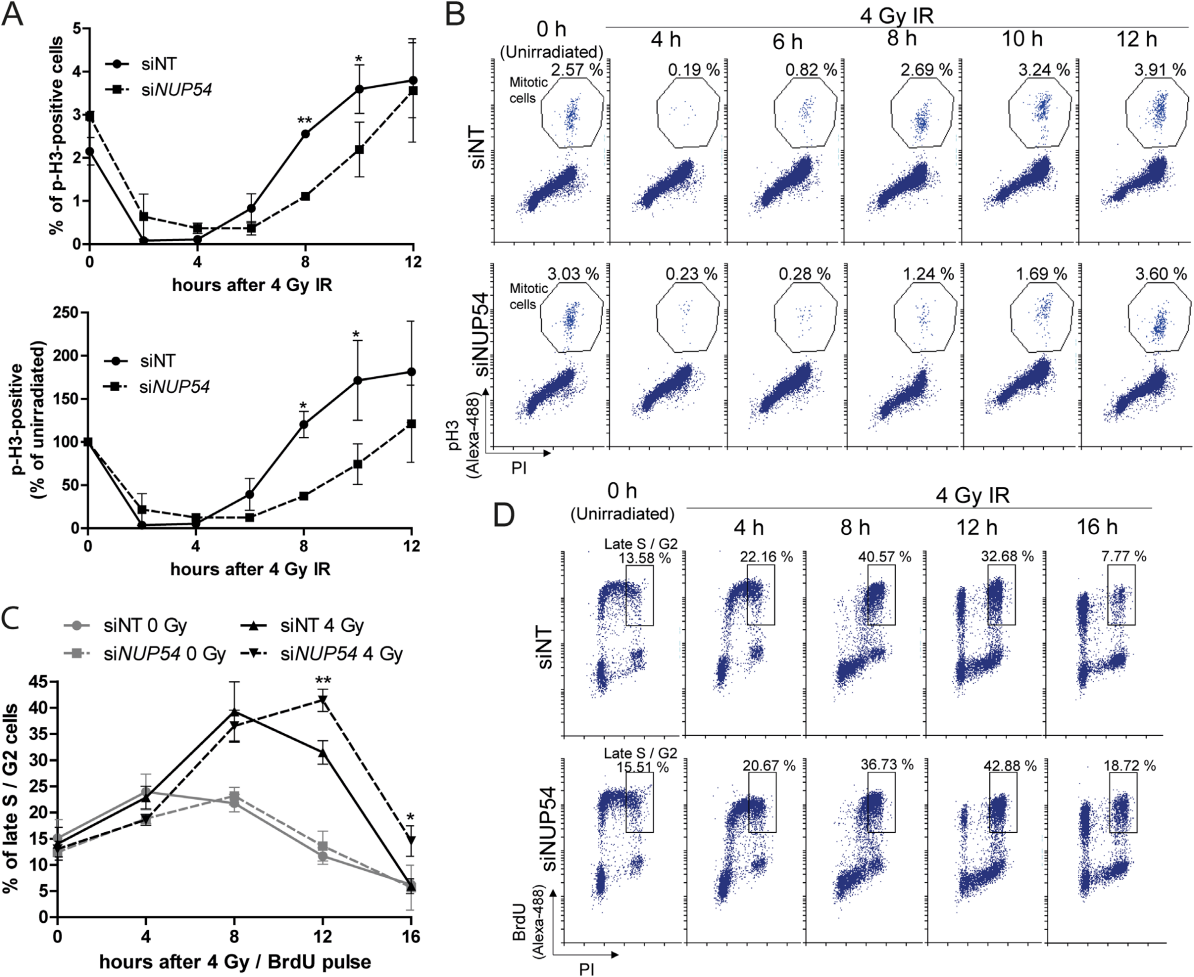
Nup54 KD induces a prolonged G2 arrest in cells irradiated in G2 and S cell-cycle phases. (**A**) Proportions of mitotic HeLa cells (cells with 4n DNA content positive for p-H3), assessed by flow cytometry, are represented as absolute percentages (top graph) or in relation to the unirradiated conditions (bottom graph). (**B**) Representative dot-plots from the experiment shown in A. (**C**) Percentage of HeLa cells in late S/G2 (cells with 4n DNA content positive for BrdU) estimated by flow cytometry. (**D**) Representative dot-plots from the experiment shown in C. Values shown in graphs correspond to mean ± sd from three independent experiments (*P < 0.05; ** *P* < 0.005; two-sided *t*-test).

DNA damage induced by IR triggers the activation of checkpoint pathways that transiently delay the progression through the G1, S and G2 cell-cycle phases, a phenomenon thought to facilitate repair and prevent cells from entering mitosis until damage is properly repaired (5,37). First, we explored potential alterations upon Nup54 KD in the progression of cells from IR-induced G2 arrest to mitosis, by assessing the proportion of cells positive for phosphorylated Histone 3 (p-H3), a mitotic marker (38). Both siNT- and si*NUP54*-treated cells showed a dramatic decrease in the proportion of mitotic cells shortly after IR, consistent with the activation of the G2 checkpoint in cells irradiated in late S/G2 (Figure 3A and B). At later time points, as cells recovered from the IR-induced G2 arrest, the proportion of mitotic cells increased. si*NUP54*-treated cells showed a delay in release from G2, with the mitotic fraction significantly lower than in siNT-treated cells at 8 and 10 h after IR exposure (Figure 3A and B). Next, we asked whether the delay in the release from IR-induced G2 arrest upon Nup54 depletion occurs in cells irradiated in S phase. For this purpose, S phase cells were pulse-labelled with bromodeoxyuridine (BrdU) immediately prior to IR treatment and the proportion of BrdU-positive cells in late S/G2 was assessed at different time points. In both siNT- and si*NUP54*-treated cells, the fraction of labelled cells in late S/G2 increased as they arrested in G2 (Figure 3C and D). In siNT-treated cells, the labelled late S/G2 fraction started to decrease after 8 h, whilst in si*NUP54*-treated cells a significantly higher proportion of late S/G2 cells was observed at 12 and 16 h after IR exposure, which suggests a delay in the progression from G2 to mitosis (Figure 3C and D).

**Figure 4.**
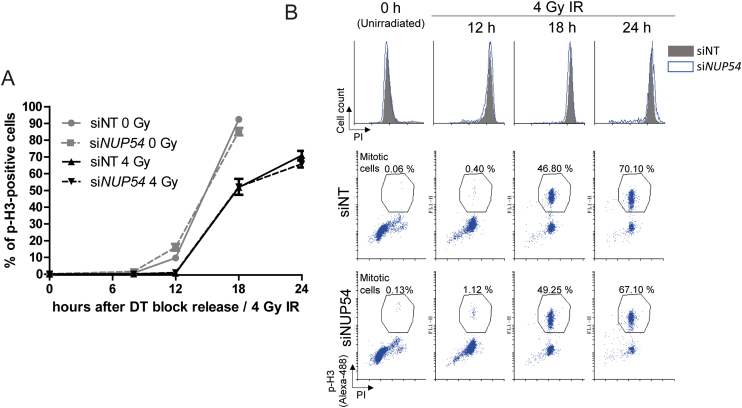
Nup54 KD does not influence the cell-cycle progression of cells irradiated in G1. (**A**) Hela cells were synchronized in G1/S by DT block, either left untreated or exposed to 4 Gy IR, and then released from the DT block and incubated with nocodazole for mitotic arrest. Cells were analysed by flow cytometry for DNA content (PI) and p-H3. Graph depicts the percentage of mitotic cells as a function of time. (**B**) Representative overlapping histograms (top panel) and dot-plots (bottom panel) from the experiment described in A. Values shown in graph correspond to mean ± sd from three independent experiments.

In order to assess any differences in the cell cycle progression of cells irradiated prior to entering replication, we synchronized cells at the G1/S boundary using a DT block. Following IR exposure, cells were released from the DT block, nocodazole was added to arrest cells in mitosis and the proportion of p-H3-positive cells was estimated at different time points (Figure 4A and B). Consistent with the activation of the DNA damage checkpoints, irradiated cells accumulated in mitosis more slowly than unirradiated cells (Figure 4A). However, no differences were observed between irradiated siNT- and si*NUP54*-treated cells. These results suggest that Nup54 depletion causes a more prolonged G2 arrest specifically in cells irradiated in S or G2, whilst it does not influence the cell-cycle progression of cells irradiated in G1.

### Nup54 depletion does not increase the radiation sensitivity of cells in G1 phase

**Figure 5.**
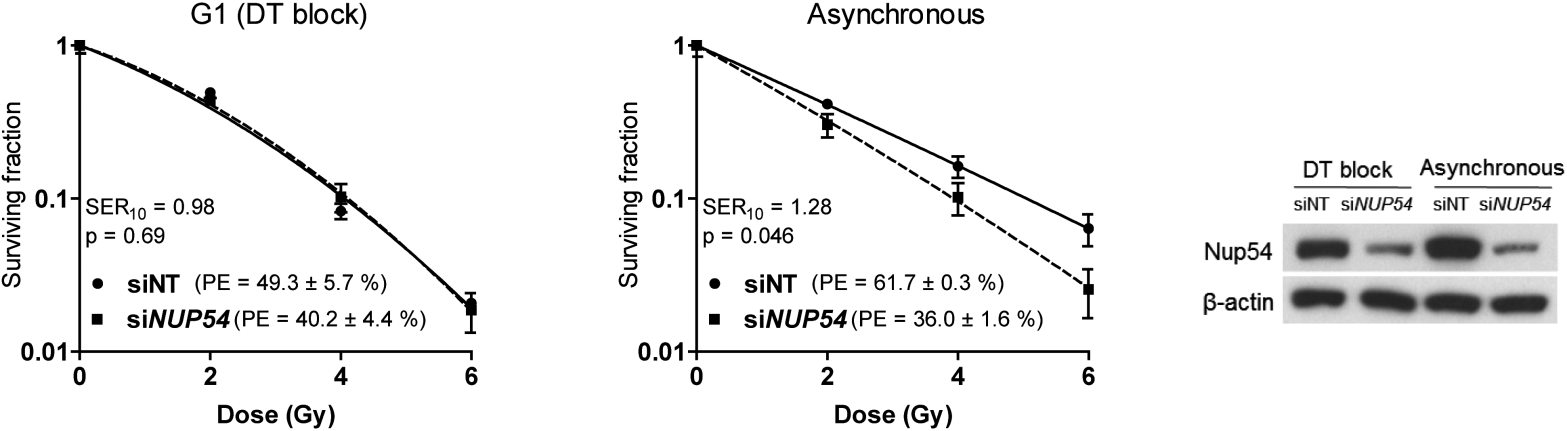
Nup54 depletion does not increase the sensitivity to IR of cells in G1. Surviving fraction assessed in CFAs in HeLa cells either synchronized in G1 or growing asynchronously at the time of IR. For G1 synchronization, cells were plated 24 h after transfection and left to attach 4 h prior initiating a DT block. Cells were released from the DT block immediately after IR exposure. For the experiment in asynchronous cultures, cells were plated 48 h after transfection and 24 h before IR. Nup54 KD at the time of IR was confirmed by western blotting. Surviving fraction, SER10 and statistical significance of differences between curves were estimated as in Figure 1.

We also examined the cell-cycle dependency of the increased sensitivity to IR upon Nup54 depletion, analysing the colony formation capability of cells synchronized at G1 phase (Figure 5). In this setting, most of the cells undergo a round of replication prior to IR, as they are seeded as single cells, left to attach and shortly after are subjected to DT block. Accordingly, a positive control for sensitization was included in parallel, in which cells were seeded 24 h prior to irradiation and were allowed to proliferate asynchronously, so these cells would also complete a round of replication. In this way, most of the colonies will be formed by two cells at the time of radiation exposure in both cases. Cell-cycle analysis confirmed near complete synchronization after DT block and an equivalent cell cycle distribution of the asynchronous cultures in both siNT- and si*NUP54*-treated cells (Supplementary Figure S2). In agreement with the cell-cycle experiments, cells irradiated at G1 did not show enhanced sensitivity upon Nup54 depletion, whilst a significant sensitization was observed in asynchronous cells (Figure 5).

### Depletion of the central channel nucleoporins Nup54, Nup62 or Nup58 does not affect the nuclear import of 53BP1

**Figure 6.**
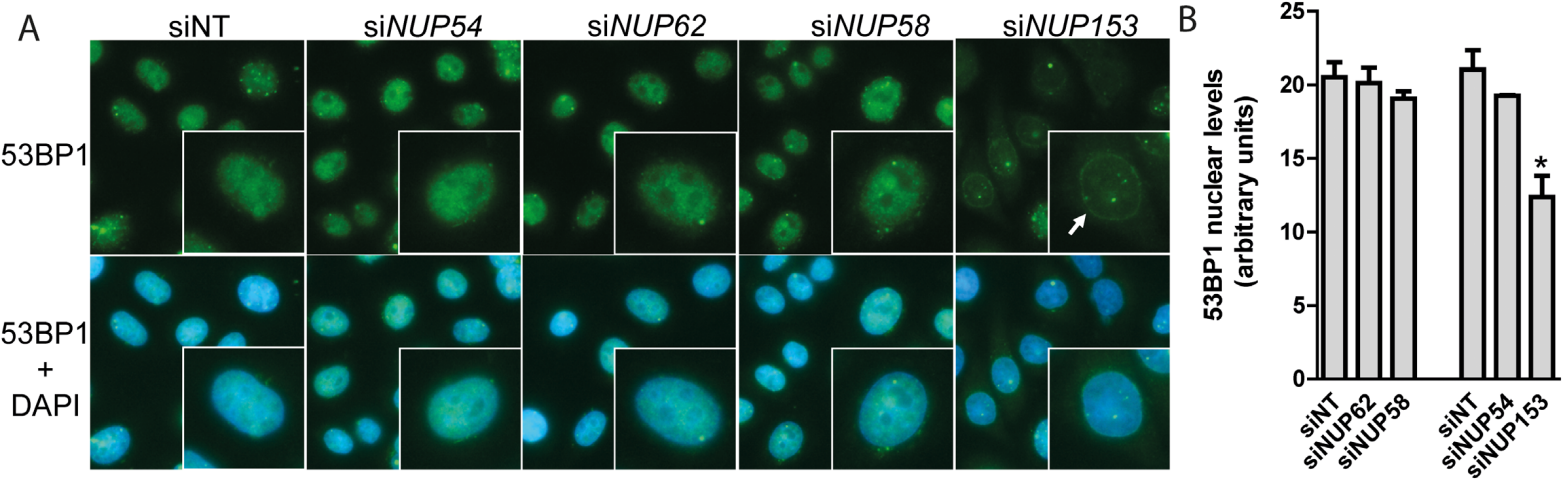
Depletion of the central channel nucleoporins Nup54, Nup62 or Nup58 does not affect the nuclear import of 53BP1. (**A**) Fluorescence microscopy images of unirradiated HeLa cells 72 h after transfection with indicated siRNAs, showing 53BP1 (green) and DNA (DAPI, blue) staining. The arrow indicates perinuclear 53BP1 accumulation. (**B**) Quantification of 53BP1 nuclear levels (mean fluorescence intensity). Values shown in graph correspond to mean ± sd (*P < 0.001, two-sided *t*-test; *n* = 3 technical replicas from a representative experiment repeated three times).

The nuclear pore basket protein Nup153 is the only nucleoporin in mammals with an established role in the response to DSBs. It has been shown that depletion of Nup153 sensitizes cells to DSB-inducing agents (phleomycin and IR) primarily by inhibiting the nuclear import of the c-NHEJ factor 53BP1 via the β-importin pathway (21,22), which is the canonical nuclear import pathway for proteins (7). To investigate whether a similar mechanism is responsible for the radiosensitization seen following Nup54, Nup62 or Nup58 depletion, we tested whether knockdown of these central channel nucleoporins affected 53BP1 nuclear levels. As previously described, Nup153 depletion caused a decrease in the levels of 53BP1 in the nucleus and a concomitant increase in the cytoplasm, with some cells displaying perinuclear accumulation, presumably at nuclear pores (Figure 6A and B). In contrast to Nup153 depletion, however, siRNA-mediated downregulation of Nup54, Nup62 or Nup58 had no impact on the 53BP1 nuclear levels. Therefore, this result suggests that the downregulation of individual central channel nucleoporins does not impair the canonical pathway for protein nuclear import and that, unlike Nup153, the radioprotective role of these nucleoporins is not exerted through the nuclear import of 53BP1.

### Nup54 depletion increases the formation of chromatid-type aberrations after IR

**Figure 7.**
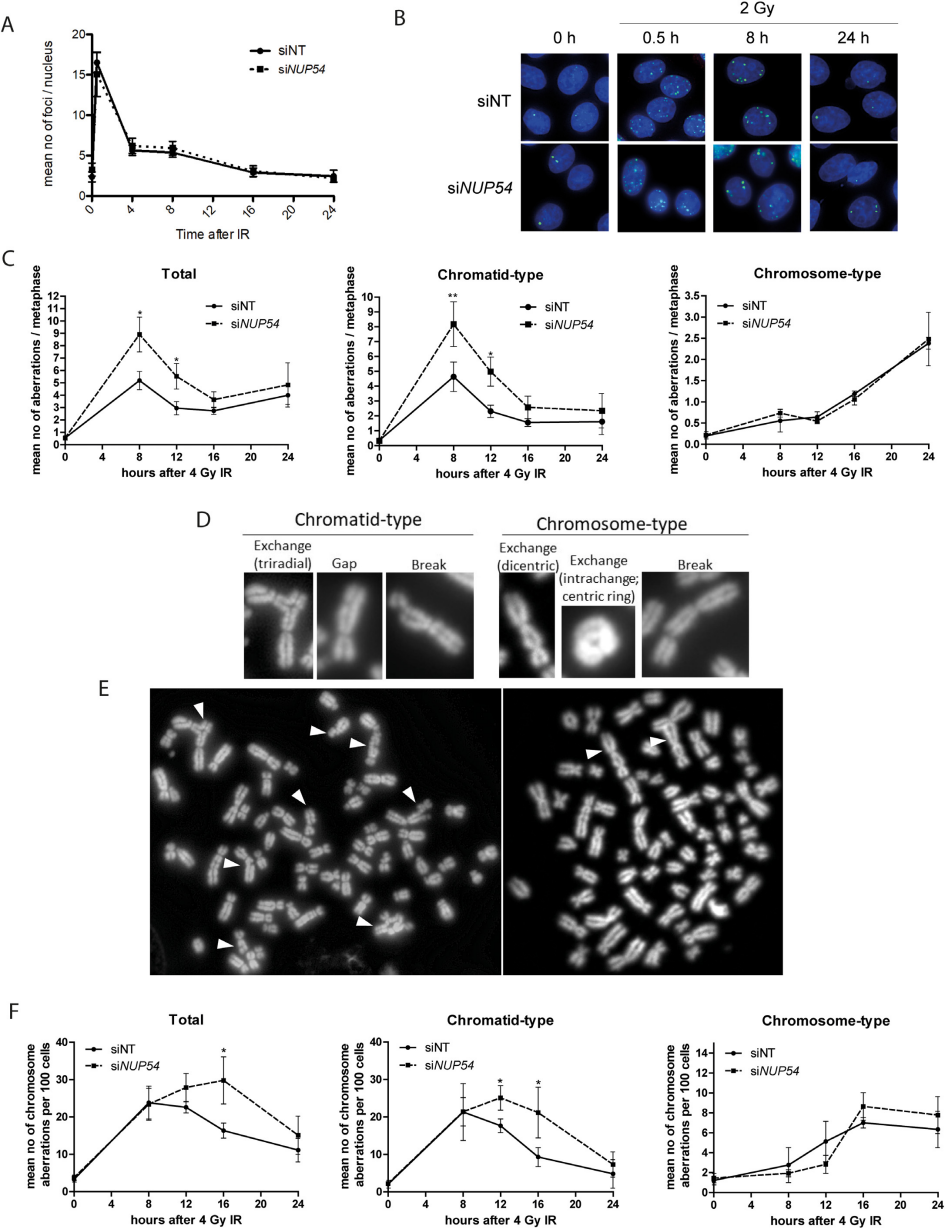
Nup54 KD increases the formation of chromatid-type aberrations after IR. (**A**) Number of γH2AX foci per nucleus in HeLa cells after 2 Gy IR (values correspond to mean ± sd and are representative of an experiment repeated three times). (**B**) Fluorescence microscopy images showing γH2AX (green) and DNA (DAPI, blue) staining in HeLa cells. (**C**) Total, chromatid-type and chromosome-type chromosome aberrations per metaphase at the indicated times after 4 Gy IR in HeLa cells. (**D**) Examples of chromatid- and chromosome-type aberration scored. (**E**) Metaphase spreads of si*NUP54*- (left) and siNT-treated (right) cells at 8 and 24 h after IR, respectively. Chromatid- (left) and chromosome-type (right) aberrations are indicated with arrows. (**F**) Mean number of chromosome aberrations per 100 cells of the total cell population, estimated by multiplying the mean number of chromosome aberrations per metaphase by the proportion of mitotic cells. Values in C and F correspond to mean ± sd from three independent experiments (*P < 0.05; ** *P* < 0.005; two-sided *t*-test).

The results presented here so far suggest that Nup54 KD causes an effect on cell cycle progression and sensitivity of cells irradiated in S and G2 phases, which might reflect the increased mitotic catastrophe observed by time lapse-microscopy. Since mitotic catastrophe occurs when cells attempt to divide in the presence of chromosome abnormalities (2), which in turn are generated as a result of either unrepaired or inaccurate re-joining of DSBs, we next asked whether Nup54 somehow influences DNA repair. We assessed the mean number of γH2AX foci per nucleus as a surrogate marker of DSBs (39) at different times after IR and found no differences between siNT and si*NUP54*-treated cells (Figure 7A and B), even when the analysis was restricted to G2 cells (Supplementary Figure S3), suggesting that Nup54 does not influence the kinetics of DSB repair.

Next we investigated whether there were any differences in chromosome aberrations. Interestingly, the analysis of chromosomes in metaphase revealed a significantly increased number of chromosome aberrations upon Nup54 depletion at 8 and 12 h after IR exposure (Figure 7C). Chromosome aberrations are either of the chromatid type—involving one chromosome arm—or the chromosome type—involving both chromosome arms. It is widely accepted that IR-induced DSBs originated in non-replicated DNA give rise mainly to chromosome-type aberrations because of the replication of the abnormality, which will appear at the same locus on both chromatids in the ensuing mitosis (Figure 7D and E) (40,41). In contrast, in replicated DNA, IR mostly causes DSBs in one of the duplicated chromosomes, so the resulting aberration will appear in just one of the chromatids in metaphase (40,41). Accordingly, the proportion of chromatid-type aberrations was higher at early time points after IR exposure, whilst chromosome-type aberrations were mostly found at late time points (Figure 7C). Of note, si*NUP54*-treated cells showed a significant increase in chromatid-type aberrations at early time-points compared to control cells, whilst no differences were found in chromosome-type aberrations at any of the time points analysed (Figure 7C). As these differences may reflect the delay in the progression of G2-arrested cells into mitosis, we estimated the number of chromosome aberrations in relation to the total cell population—mean number of aberrations per 100 cells—by multiplying the percentage of mitotic (p-H3-positive) cells by the mean number of chromosome aberrations per metaphase. We confirmed that Nup54 depletion causes an increase in the absolute number of both total and chromatid-type chromosome aberrations at different time points after IR (Figure 7F), whilst it does not lead to significant differences in the absolute number of chromosome-type aberrations. These findings suggest that Nup54 protects against the formation of chromosome aberrations arising from DSBs generated in replicated DNA, and are in agreement with the prolonged arrest and increased radiosensitivity found specifically in cells that contained replicated DNA at the time of IR exposure.

### Nup54 is required for the efficient repair of DSBs by HR

**Figure 8.**
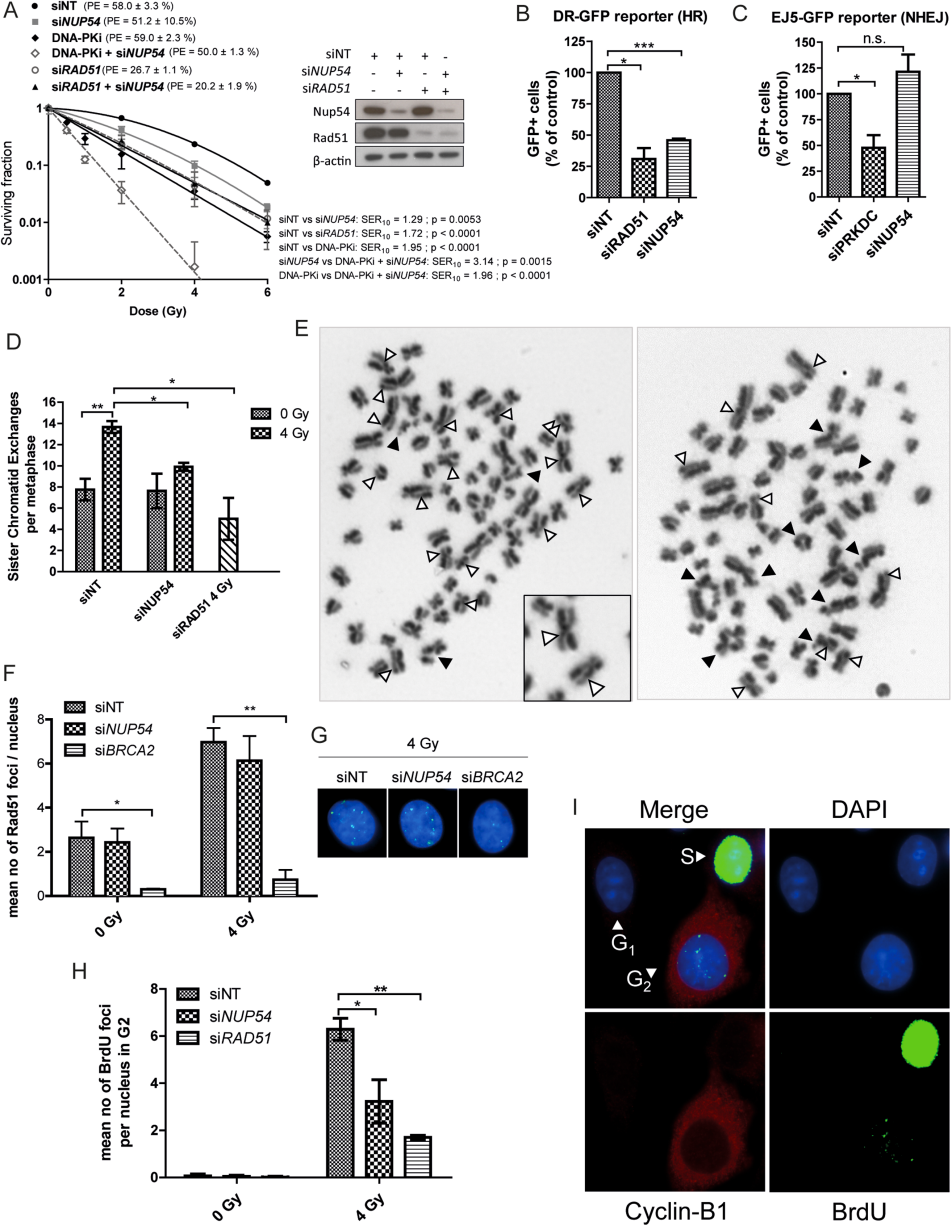
Nup54 depletion does not further radiosensitize HR-deficient cells and impairs HR-mediated repair. (**A**) CFA of HeLa cells transfected with siNT, si*NUP54* and si*RAD51*, alone or in combination (40 nM siNT (siNT); 20 nM siNT + 20 nM si*NUP54* (si*NUP54*); 20 nM siNT + 20 nM si*RAD51* (si*RAD51*); 20 nM si*NUP54* + 20 nM si*RAD51* (si*RAD51* + si*NUP54*)). One hour before IR exposure, 1 μM NU7441 (DNA-PK inhibitor (DNA-PKi)) was added to siNT and siNT + si*NUP54*-treated cells (DNA-PKi and DNA-PKi + si*NUP54*, respectively), and removed 24 h after IR exposure. Surviving fraction, SER_10_ and statistical significance of differences between curves were estimated as in Figure 1. (**B**) HR repair efficiency determined with the DR-GFP reporter in HEK293 treated with the indicated siRNAs. si*RAD51* was used as a positive control. (**C**) NHEJ repair efficiency determined with the EJ5-GFP reporter in HEK293 treated with the indicated siRNAs. An siRNA targeting DNA-PK (si*PRKDC*) was used as a positive control. (**D**) Mean number of SCEs per metaphase in HeLa cells accumulated in mitosis from 8 to 12 h after 4 Gy IR. (**E**) Examples of metaphase spreads of siNT- (right) and si*NUP54*-treated (left) cells. SCEs and chromatid-type aberrations are indicated with white and black arrows, respectively. Examples of SCEs are shown at a higher magnification (inset). (**F**) Mean number of Rad51 foci per nucleus in HeLa cells at 6 h after IR. si*BRCA2* was used as a positive control for Rad51 foci formation inhibition. (**G**) Image of Rad51 foci in siNT- and si*NUP54*-treated cells 6 h after 4 Gy IR (green: Rad51; blue: DAPI). (**H**) Mean number of BrdU foci detected after DNase treatment in HeLa cells irradiated with 4 Gy in G2 phase (6 h after IR). (**I**) Staining pattern of cells from experiment depicted in H. With the exception of graph shown in A, values correspond to mean ± sd from three independent experiments (*P < 0.05; **P < 0.005; ***P < 0.001; n.s.: non-significant; two-sided *t*-test).

Since Nup54 influences the cell cycle progression and radiosensitivity specifically in cells irradiated in S and G2, and considering that HR repair is restricted to these cell-ycle phases (6), we asked whether the protective role of Nup54 against IR could be dependent on this pathway. Combined siRNA-mediated downregulation of the HR factor Rad51 (6) and Nup54 did not result in a further increase in radiosensitivity when compared to the individual KD of the two proteins separately (Figure 8A). In contrast, inhibition of DNA-PK, an essential factor in the c-NHEJ repair pathway (6), which is active throughout all the cell cycle phases, further increased the sensitivity of si*NUP54*-treated cells to IR (Figure 8A). Therefore, a competent HR repair pathway seems to be required for Nup54 depletion to cause an increase in the sensitivity to radiation, suggesting that Nup54 may have a role in HR repair. To further support this observation, we evaluated the repair efficiency of different repair pathways using previously described chromosome-integrated reporters in HEK293 cells (26). Such reporters are based on the restitution of a GFP expression cassette by the action of a particular repair pathway on DSBs generated by the I-SceI endonuclease, which can be scored using flow cytometry (26). siRNAs targeted against Rad51 and DNA-PKcs were used as positive controls for the inhibition of HR (DR-GFP reporter) and total NHEJ (EJ5-GFP reporter) activity, respectively (Figure 8B and C). Nup54 depletion caused a marked reduction in the efficiency of HR repair, at levels close to the ones achieved with si*RAD51* (Figure 8B). Conversely, Nup54 depletion caused a slight, albeit not significant, increase in the NHEJ repair activity (Figure 8C).

In order to provide more evidence on the role of Nup54 in HR, we scored the recombinogenic events at DSBs sites resolved with crossover using the SCE assay (42). In this assay, chromatid exchanges are visualized as reciprocal discontinuities in the differential staining of sister chromatids (Figure 8D and E). Nup54 depletion did not produce any significant variation in the number of SCEs in unirradiated cells, but strongly inhibited SCE formation in metaphases accumulated between 8 and 12 h after 4 Gy IR, as with Rad51 depletion (Figure 8D). We confirmed a simultaneous increase in the formation of chromatid-type aberrations (Supplementary Figure S4), consistent with the notion that this type of aberration occurs as a consequence of failed recombinogenic repair events.

Since Nup54 depletion did not seem to impair Rad51 foci formation (Figure 8F and G), we asked whether Nup54 could be involved in a more downstream step. One possibility could be the DNA synthesis across the resected DNA strand that occurs after Rad51-mediated strand invasion and annealing with the homologous sequence (6). This extensive DNA synthesis distinctive of the HR repair pathway can be monitored by fluorescence microscopy combining BrdU labelling and a G2 phase marker (e.g. CENP-F, Cyclin-B1), as previously described (Figure 8H and I) (30). Aphidicolin is added to prevent progression from S to G2 phase and cells are incubated with BrdU during the repair time, immediately after IR. S phase cells will appear with a strong pan-nuclear BrdU staining, G2 cells will be positive for the G2 marker and pan-nuclear BrdU-negative, whilst G1 cells will be double-negative (Figure 8I). After DNase treatment, sites of DNA synthesis linked to HR repair are evident in the nucleus of G2 cells as foci of BrdU incorporation (Figure 8I). As shown in Figure 8H, Nup54 depletion considerably inhibited BrdU foci formation in G2 cells. A strong decrease was also seen in the si*RAD51*-treated cells used as a positive control. Therefore, Nup54 seems to be required for efficient DNA synthesis linked to the repair of DSBs by HR in cells irradiated in G2 phase.

Altogether, these results demonstrate that Nup54 contributes to the efficient repair of DSBs mediated by HR.

## DISCUSSION

In the present study, using a high-throughput colony formation screen, we identified the NPC protein Nup54 as a novel factor involved in the protection against IR. A similar role was found for Nup62 and Nup58, the other nucleoporins which along with Nup54 constitute the central channel of the NPC (34,35). Interestingly, Nup54 depletion only appeared to affect cells that contained replicated DNA at the point of irradiation, where it increased the duration of the subsequent IR-induced G2 arrest and the sensitivity to IR. In agreement with this cell cycle selectivity, we found that Nup54 depletion increases the formation of chromatid-type aberrations, which are known to arise mostly from DSBs originated in replicated DNA (40,41). Conversely, we found that Nup54 depletion does not influence the formation of chromosome-type aberrations, which arise mostly from DSBs originated in non-replicated DNA (40,41). Moreover, we demonstrated that Nup54 depletion increases cell death by mitotic catastrophe following IR exposure, which occurs when a cell containing lethal chromosome alterations attempts to go through mitosis (2). Altogether, these results suggest that Nup54 contributes to the protection from cell death caused by DSBs and to the maintenance of the structural integrity of chromosomes.

Despite the increased presence of chromatid-type chromosome aberrations in irradiated cells depleted of Nup54, we found no differences in the dynamics of DSB repair, as shown by γH2AX foci analysis. Although this might seem surprising particularly in the case of chromatid terminal deletions—where there is a visible discontinuity in the chromatin—there is compelling experimental evidence demonstrating that the presence of chromatid breaks in metaphase does not depend on the DSB re-joining rate (41,43). Whilst the mechanism of chromatid break formation is still a subject of debate (40,41,43,44), it is broadly accepted that exchange-type aberrations form when two break ends from different chromosomes are erroneously ligated (1,45,46). Both c-NHEJ and HR are known to prevent the formation of chromatid-type aberrations in cells irradiated in S and G2 phases (40,47). Interestingly, we provide evidence demonstrating that Nup54 is required for the efficient repair of DSBs specifically through the HR pathway. We found that Nup54 depletion does not affect the radiosensitivity of cells with impaired HR repair (upon Rad51 depletion) but it does increase the radiosensitivity of cells with impaired c-NHEJ repair (in the presence of DNA-PKi). Furthermore, in our reporter experiments, we observed that Nup54 KD decreases the HR repair activity but does not affect NHEJ. Finally, we demonstrated that Nup54 depletion decreases the formation of SCEs and DNA synthesis foci in G2 cells after IR, both processes linked to the repair of DSBs by HR (30,42). In view of these results and since Nup54 depletion did not affect Rad51 foci formation, we hypothesize that Nup54 is involved downstream of Rad51 binding to DNA.

A role of Nup54 in HR repair is also consistent with our finding that Nup54 depletion increased the formation of chromatid-type aberrations, as well as the sensitivity and the duration of the G2 arrest in cells irradiated in S and G2, the cell-cycle phases where HR takes place (6). Moreover, considering that c-NHEJ is the main DSB repair pathway throughout the cell cycle (6), these findings also agree with our observation that Nup54 KD did not affect the cell cycle and the sensitivity of cells irradiated in G1, or the formation of chromosome-type aberrations. A defect in post-replicative DNA/HR repair upon Nup54 depletion could also account for the different extent of the delay in the progression of cells irradiated in G2 into mitosis compared to that of cells irradiated in S phase. The observation that the latter was less pronounced agrees with the lower quantity of replicated DNA in S phase compared to G2 phase cells.

c-NHEJ is thought to efficiently avoid formation of exchanges between chromosomes through its capability to rapidly stabilize and repair DSBs (1,46). On the other hand, the ability of HR to prevent chromatid exchanges between different chromosomes lies on its high preference for sister chromatids as homologous templates (1,46). Inhibition of HR repair, as upon Rad51 depletion, determines the activation of the DNA-PK-independent alt-EJ or the SSA repair pathways (6,46–50). SSA and alt-EJ are more prone to repair errors and the formation of chromosome aberrations than HR and c-NHEJ (46,47,51). It could therefore be speculated that an increase in the repair activity of the alt-EJ and SSA pathways may be behind the increase in chromatid-type aberration formation in Nup54-depleted cells after radiation. The extreme radiation sensitivity observed in Nup54-depleted cells upon DNA-PK inhibition could also indicate that Nup54 contributes to the repair activity that may act as a backup for c-NHEJ, as might be expected from a HR repair factor (48).

Although several NPC sub-complexes have been implicated in the response to DSBs in yeast, the nuclear basket is the only one to have been previously linked to DSB repair in mammals (21–25). Whilst yeast NPCs seem to actively recruit DSBs (10–18), this mechanism is unlikely to happen in mammalian cells, in which DSBs do not migrate to the nuclear periphery (19,20). In fact, mammalian nuclear basket nucleoporins seem to promote DSB repair by allowing the import of 53BP1 via the importin-β pathway (through Nup153) (21–23), as well as its recruitment to DNA damage foci (mediated by both Nup153 and Nup50) (25). Therefore, although NPCs seem to have an evolutionary conserved role in the protection against DSBs, the mechanisms of action may substantially differ. Nup54, Nup62 and Nup58 contain phenyl-glycine (FG) repeat motifs that are thought to restrict the transit of large macromolecules throughout the NPC, thus constituting a selectively permeable barrier (34,35,52). Although not all reports are in agreement, the current literature suggests that individual central channel nucleoporins are required for efficient functioning of mRNA export and protein import/export pathways, including the importin-β pathway (52–56). In the present work, however, Nup54, Nup62 or Nup58 depletion was not associated with impaired 53BP1 import, demonstrating that the radioprotective role of central channel nucleoporins is not due to major import defects and, in agreement with a specific role of Nup54 in HR repair, that it is not exerted through the Nup153/importin-β/53BP1 pathway. It is possible, however, that the KD of central channel nucleoporins selectively affects the import of certain proteins and not others (22,57).

It would therefore be interesting to explore whether the role uncovered here for Nup54 is mediated through a specific nucleocytoplasmic transport pathway, presumably affecting the location of a HR-related factor. Alternatively, the role of Nup54 in the response to DSBs could be exerted through mechanisms other than the regulation of nucleocytoplasmic trafficking (58,59). Particular attention should be paid to HR-related processes that may avoid illegitimate joining of breaks and the emergence of chromatid-type aberrations. In this regard, mammalian nucleoporins are known to contribute to the spatial organization of chromatin (58) and, in turn, it is known that chromatin organization can influence repair pathway choice (60) and the formation of chromosome aberrations (40,45). Moreover, given the emerging role of nucleoporins as transcriptional regulators (59), expression regulation of factors involved in HR should also be considered as a potential mechanism explaining the radioprotective role of Nup54.

The role revealed here for Nup54 could be relevant from a translational perspective. Mutations in HR genes (e.g. *BRCA1* and *BRCA2*) and their epigenetic status (e.g. *BRCA1* silencing) have been linked to an increased risk of developing a variety of cancers (61,62). Deficiencies in HR repair factors are associated with a high sensitivity not only to IR, but also to several other DNA damaging agents and drugs, and are currently exploited in the clinic to guide treatment decisions (61–63). Specifically, poly (ADP-ribose) polymerase (PARP) inhibitors are an established and effective treatment for patients with tumours harbouring BRCA mutations (63). A major limitation for the use of known HR deficiencies as biomarkers is the low frequency with which they are found in cancer patients (62). In this regard, the discovery of new factors involved in HR could help to better define the DNA repair capability of tumours and tailor targeted therapies more appropriately. Moreover, at the moment there are no specific HR inhibitors, with the exception of few Rad51 inhibitors which have not been tested in a clinical setting yet (63). It could therefore be very interesting to evaluate the suitability of Nup54 as a biomarker and a therapeutic target in cancer.

## CONCLUSION

In summary, our study adds Nup54, Nup62 and Nup58 to the list of NPC proteins with a role in the DNA damage response. Our results suggest that Nup54 contributes to the protection of cells from DSB-induced cell death and safeguards the integrity of the genome. Nup54 seems to modulate the DNA damage response in cells irradiated in S and G2 and, accordingly, is required for efficient repair of DSBs specifically through the HR repair pathway.

## Supplementary Material

Supplementary Data
