# Supplementary material for: Nucleoporin 54 contributes to homologous recombination repair and post-replicative DNA integrity
Source: Nucleic Acids Res. 2018 Jul 9;46(15):7731–46. doi: 10.1093/nar/gky569 (PMC6125679; doi:10.1093/nar/gky569)
Supplement: Supplementary Data [file gky569_supplemental_files.zip › Supplementary Figures_Rodriguez-Berriguete et al., 2018.pdf]

Supplementary Figures

Nucleoporin 54 contributes to Homologous Recombination repair and post-replicative DNA integrity

Gonzalo Rodriguez-Berriguete, Giovanna Granata, Rathi Puliyadi, Gaganpreet Tiwana, Remko Prevo, Sheng Yu, Francesca Buffa, Timothy C. Humphrey, W. Gillies McKenna and Geoff S. Higgins

CRUK/MRC Oxford Institute for Radiation Oncology, University of Oxford, Old Road Campus Research Building, Roosevelt Drive, Oxford OX3 7DQ, UK

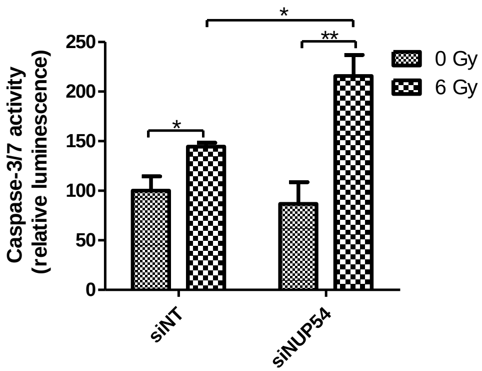

Supplementary Figure S1. Nup54 depletion potentiates the induction of caspase-3/7 activity by IR. 72 hours after transfection with the indicated siRNAs HeLa cells were seeded in white-walled 96 well-plates (3 x 10^4 cells / well) and left to attach for 4 hours before applying 6 Gy IR. 72 hours later, medium was removed and cells were incubated with 50 µL of 10 µg/mL resazurin (SIGMA) for 1 hour. The corresponding fluorescence signal was measured with a plate reader (PolarStar, Omega) and used as an estimate of cell density. Afterward, 50 µL of caspase-Glo 3/7 reagent (Promega) were added and plates were incubated for 2 hours at room temperature. Luminescence was measured using the plate reader, normalised for cell density and represented in relation to control wells (*p < 0.05. **p < 0.005; two-sided t-test; n = 4 technical replicas; two independent experiments showing similar results).

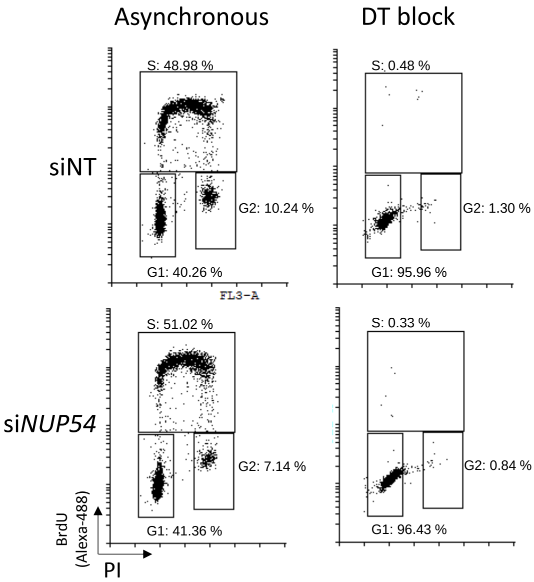

Supplementary Figure S2. Cell cycle distribution at the time of IR of HeLa cells either growing asynchronously or subjected to DT block, from experiment shown in Figure 5. Cells were incubated with 10 µM BrdU for 30 min before fixation. Samples were processed as described in Material and Methods for the BrdU label-chase experiment.

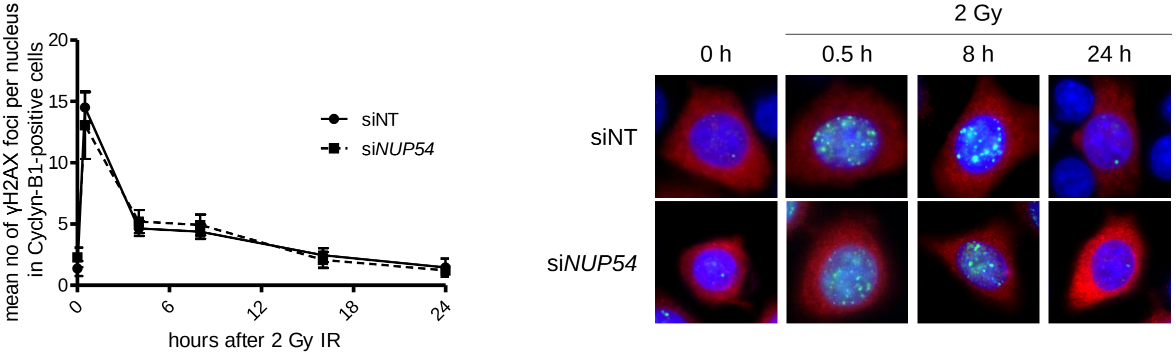

Supplementary Figure S3. γ-H2AX foci kinetics in Cyclin-B1-positive cells after 2 Gy IR. Values correspond to mean ± sd and are representative of an experiment repeated three times. Blue: DAPI; red: Cyclin-B1; green: γH2AX.

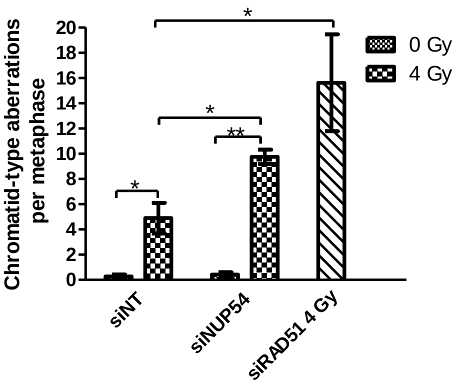

Supplementary Figure S4. Chromatid-type aberrations scored in the SCE experiment shown in Figure 7D, confirming the increased formation of this type of aberration with Nup54 depletion after IR (* $p < 0.05$. ** $p < 0.005$; two-sided t-test; $n = 3$ independent repeats).
